# Radiotherapy-induced changes in corneal morphology and biomechanics in myopathic thyroid eye disease

**DOI:** 10.3389/fmed.2025.1612518

**Published:** 2025-06-03

**Authors:** Pengsen Wu, Jing Rao, Shenglan Yang, Xiaohan He, Yuqing Wu, Guiqin Liu

**Affiliations:** ^1^Shenzhen Eye Hospital, Shenzhen Eye Medical Center, Southern Medical University, Shenzhen, China; ^2^Shenzhen OCT Hospital, Shenzhen, China

**Keywords:** thyroid eye disease, radiotherapy, corneal morphology, biomechanics, Corvis ST

## Abstract

**Purpose:**

To assess differences in corneal morphology and biomechanics between patients with myopathic thyroid eye disease (TED) and normal controls (NCs), as well as to evaluate these changes following radiotherapy.

**Methods:**

Patients with active myopathic TED and age-, sex-, and spherical equivalent-matched NCs were enrolled in this study. All patients underwent radiotherapy and were followed up for 6 months post-treatment. Complete ophthalmic examinations were conducted for all subjects. Corneal morphology was evaluated using Pentacam, and biomechanics were assessed with Corvis ST tonometry, both before and 6 months post-radiotherapy.

**Results:**

This prospective, comparative clinical study involved 50 eyes from 50 TED patients and 30 eyes from 30 NCs. Post-radiotherapy, the intraocular pressure (IOP), proptosis, disease activity, diplopia, eye motility restriction and muscular index in TED patients significantly improved. Corneal morphological parameters such as elevation deviation, including back elevation (Db), pachymetric progression, relational thickness, overall deviation, and the index of surface variance, were significantly greater in TED patients compared to NCs, while Ambrosio’s Relational Thickness maximum was significantly lower. After radiotherapy, Db decreased, and posterior surface astigmatism increased with statistical significance. Significant biomechanical changes were observed in patients with TED after adjustment with biomechanically corrected IOP, including reduced A2 time and peak distance, increased tomographic and biomechanical index (TBI), as well as diminished maximum whole eye movement length (WEML). Post-radiotherapy, a significant increase in WEML was observed. Correlation analysis suggested that improvements in ocular biomechanics may be linked to reductions in IOP and proptosis.

**Conclusion:**

Patients with myopathic TED demonstrated observable corneal alterations, including increased steepness, surface irregularity, reduced corneal hysteresis, and decreased orbital compliance. These biomechanical changes were associated with elevated TBI values, indicating heightened susceptibility to corneal ectatic disorders. Ocular biomechanical parameters may serve as as potential quantitative biomarkers for assessing treatment effectiveness in TED management.

## Introduction

Thyroid eye disease (TED), also referred to as Graves’ ophthalmopathy (GO), is the most prevalent extrathyroidal condition related to Graves’ disease (GD). Approximately 20%–25% of GD patients experience clinically significant TED, with 3%–5% progressing to severe TED (1). TED is a pathologic autoimmune orbital disease characterized by diffuse lymphocytic infiltration of the extraocular muscles (EOMs) and periorbital connective tissues, leading to the enlargement of these structures (2). Treatment options are determined by disease severity and activity. Mild TED might improve spontaneously, whereas moderate to severe and active TED requires either medical or surgical treatment. High-dose intravenous administration of glucocorticoids is the first-line therapeutic regime for moderate to severe active TED; however, approximately 20%–40% of patients might not respond effectively (3, 4). Retro-orbital radiotherapy is recommended as one of the second-line treatment choices, especially for myopathic TED patients with diplopia and/or restricted eye movement. By suppressing lymphocyte infiltration and fibroblast proliferation, as well as glycosaminoglycan (GAG) secretion, radiotherapy provides non-specific anti-inflammatory effects to alleviate orbital pressure and local symptoms (5–7).

Previous studies have identified distinct alterations in corneal morphology and biomechanics in patients with TED (8–10). Notably, recent evidence suggests that TED patients exhibit characteristic corneal abnormalities resembling keratoconus, accompanied by elevated anterior corneal astigmatism along the oblique axis (8). Comparative analyses further demonstrated significantly reduced orbital compliance and impaired biomechanical parameters in active TED patients compared to healthy controls (11, 12). These findings highlight the potential utility of corneal parameters as diagnostic markers for early-stage TED detection. Furthermore, growing research suggests a potential association between TED and corneal ectatic disorders. A recent case report documented TED-induced progression of secondary keratoconus (13), while a cross-sectional study identified thyroid dysfunction as a possible risk factor for corneal ectasia (14). However, the relationship between TED and corneal morphology and biomechanics remains to be further explored.

Treatment of TED can alter corneal biomechanics. For moderate to severe active TED patients, intravenous glucocorticoids can improve orbital compliance as evidenced by improved whole eye movement obtained using a dynamic Scheimpflug analyzer (Corvis ST) (15). In stable TED, orbital decompression surgery leads to a decrease in ocular biomechanics, whereas corneal stiffness and orbital tissue compliance tend to increase following anterior blepharotomy (9, 10). In addition, strabismus surgery for TED may affect corneal topography by changing the tension of EOMs or altering intraocular pressure (IOP) (16). While radiotherapy has demonstrated efficacy in reducing EOM volume and improving ocular motility restrictions (17, 18), its potential effects on ocular biomechanics remain unexplored, with no reported data currently available. This study aims to evaluate the corneal morphological and biomechanical changes in patients with TED, as well as to assess radiotherapy-induced alterations in these parameters.

## Materials and methods

This prospective clinical study enrolled patients with myopathic TED who were recruited from Shenzhen Eye Hospital between August 2023 and July 2024. Healthy individuals, matched in terms of age, sex, and spherical equivalent (SE), were enrolled simultaneously. The diagnosis of TED was made according to the European Group on Graves’ Orbitopathy (EUGOGO) consensus statement (3). The inclusion criteria were as follows: ① Age between 30 and 70 years; ② SE of less than 6.0 diopters; ③ Diagnosis of active TED with diplopia or restricted eye movement, confirmed by CT or MRI showing EOM enlargement; and ④ No corticosteroid medications, biologic agents, or immunosuppressive therapies for at least 3 months before the study. The exclusion criteria included: ① Systemic diseases, including other autoimmune disorders, hypertension, or diabetes; ② History of ophthalmic conditions such as glaucoma, diabetic retinopathy, or uveitis; ③ Previous ocular surgeries or trauma; and ④ History of contact lens use. The disease activity of TED was assessed according to the EUGOGO guidelines (3).

### Retro-orbital radiation

All patients underwent three-dimensional conformal radiotherapy with a 6 MV linear accelerator. The clinical target volume (CTV) encompassed the bilateral retrobulbar tissues, including the EOMs and retrobulbar fat. A 2 mm extension in all directions was applied to the CTV to establish the planning target volume. A total of 20 Gy was delivered to the patients in 10 fractions over 2 weeks. To prevent transient exacerbations of ocular manifestations during irradiation, patients were administered a concurrent low dose of 40 mg of oral prednisone daily for 2 weeks.

### Ophthalmic examination

Comprehensive ophthalmic examinations, including best-corrected visual acuity (BCVA), IOP, eye globe movement, slit-lamp biomicroscopy, Hertel exophthalmometry, and autorefraction (Topcon, Inc., Tokyo, Japan) were conducted before radiotherapy and 6 months afterward. The VISA classification system was used to assess diplopia and ocular motility restriction (19, 20). Diplopia was classified as follows: 0 = none, 1 = diplopia with gaze, 2 = intermittent diplopia, and 3 = constant diplopia. For motility restriction, the VISA grades were as follows: 0 for duction > 45°, 1 for 30–45°, 2 for 15–30°, and 3 for ≤ 15°. The strabismus angle was measured with the synoptophore.

### Muscular index measurements

Magnetic resonance imaging (MRI) or computed tomography (CT) was conducted to assess changes in the EOMs using standardized orbital imaging protocols. In TED, muscle thickening primarily affects the muscle belly. Based on previous literature reports, we measured the maximal thickness of the EOMs at 0.5 cm posterior to the globe’s posterior pole, which is the characteristic site of maximal muscle hypertrophy in TED (21). At this standardized location, we delineated the boundaries of both the EOMs and the bony orbit on coronal sections to obtain precise cross-sectional area measurements. The muscular index was then calculated as the ratio of the rectus muscle cross-sectional area to the corresponding bony orbital area (22).

### Corneal morphological measurements

The corneal topography of each patient was assessed using a Scheimpflug device (Pentacam; Oculus, Wetzlar, Germany). The topographic parameters included measurements of flat keratometry (K1) and steep keratometry (K2) on both the anterior and posterior surfaces, along with astigmatism, thinnest corneal thickness, corneal thickness at the apex, and maximum keratometry on the sagittal front (Kmax). The Belin/Ambrósio Enhanced Ectasia Display (BAD) software was utilized to illustrate the elevation deviation from normality, encompassing deviations from normal in terms of front elevation (Df), back elevation (Db), pachymetric progression (Dp), thinnest corneal point (Dt), and relational thickness (Da), along with the overall deviation from normality (D). Additional parameters, including the index of surface variance (ISV), index of vertical asymmetry (IVA), keratoconus index (KI), center keratoconus index (CKI), index of height asymmetry (IHA), and index of height decentration (IHD), were used to evaluate the shape and irregularity of the cornea.

### Corneal biomechanical measurements

Corneal biomechanical properties were assessed using the Corvis ST tonometer (Oculus, Wetzlar, Germany), which measures dynamic response parameters such as temporal characteristics [A1 time, A2 time, and highest concavity (HC) time], velocity parameters (A1 velocity and A2 velocity), deformation metrics [peak distance, HC deformation amplitude, HC deflection amplitude, and deformation amplitude ratio at 2 mm (DA ratio)], structural indices [integrated radius and stiffness parameter A1 (SP-A1), reflecting corneal resistance to deformation], and clinical composites [biomechanically corrected intraocular pressure (bIOP)]. Additionally, orbital compliance parameters, including maximum whole eye movement length (WEML) and time (WEMT), were also measured. Furthermore, two validated diagnostic indices - the Corvis Biomechanical Index (CBI) and Tomographic/Biomechanical Index (TBI) - were calculated through multivariate analysis of dynamic corneal response parameters, both demonstrating high discriminative power for differentiating normal from keratoconic corneas.

### Statistical analysis

Given the variable clinical presentation of TED, which may manifest with either unilateral or bilateral involvement. To ensure methodological consistency in statistical analyses, the affected eye was included for patients with unilateral TED. For bilateral cases, the more severely affected eye was selected for analysis. For normal controls (NCs), one eye was randomly chosen for inclusion. SPSS (version 25, IBM Corp., NY, United States) was used to conduct the analysis, and the results are presented as mean ± standard deviation (SD). For the analysis of continuous data between the TED group and the NC group, the student’s *t*-test was employed for normally distributed data, whereas the Mann–Whitney test was utilized for non-normally distributed data. For the analysis of normally distributed data between TED patients before and after radiotherapy, a paired *t*-test was performed, while the Wilcoxon signed-rank test was used for non-normally distributed data. The chi-square test was used to analyze the categorical data. Furthermore, analysis of covariance (ANCOVA) was conducted, with IOP as a covariate, to compare changes in biomechanical parameters between groups. Correlations between the two groups were assessed using either Pearson or Spearman correlation analysis, depending on data type and distribution, with adjustments for potential confounding factors. A *p*-value of less than 0.05 was considered statistically significant.

## Results

### General clinical characteristics and baseline data

Fifty patients (50 eyes) with myopathic TED and a control group of 30 eyes from 30 healthy participants were included in this study. There were no differences in sex distribution, age, or SE between the TED patients and healthy subjects, as shown in Table 1.

**TABLE 1:** Demographic data of TED patients and healthy controls.

	TED	Healthy control	*P*-value
No. of participants	50	30	
Sex, Male/Female	32/18	14/16	0.13^a^
Age (year)	46.4 ± 9.36	43.13 ± 7.12	0.1^b^
CAS	3.1 ± 0.99	/	/
Duration of thyroid disease (month)	38.6 ± 64.7	/	/
Duration of TED (month)	8.48 ± 12.12	/	/
Hyperthyroid, n (%)	10 (20%)	/	/
Hypothyroid, n (%)	9 (18%)	/	/
Eurhyroid, n (%)	31 (62%)	/	/
History of I131 treatment, n (%)	9 (18%)	/	/
History of thyroid surgery, n (%)	6 (12%)	/	/
History of glucocorticoid therapy, n (%)	23 (46%)	/	/
Spherical equivalent (D)	−0.83 ± 1.85	−1.13 ± 1.31	0.09^c^

TED, thyroid eye disease; CAS, clinical activity score.

*^a^*Chi-square test;

*^b^*Unpaired Student’s *t*-test;

*^c^*Man-Whitney test.

### Clinical outcomes of the TED patients before and 6 months after radiotherapy

**FIGURE 1:**
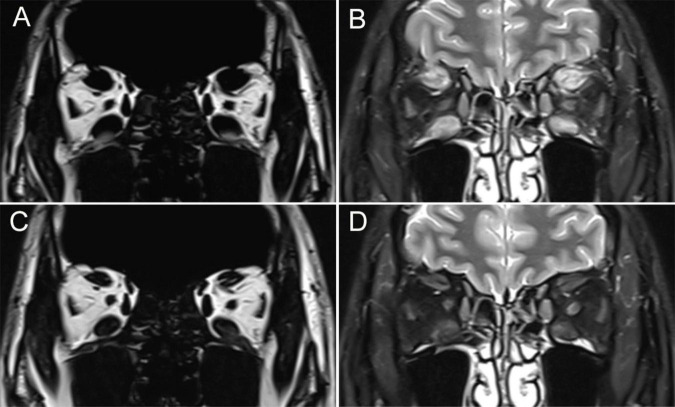
Representative MRI findings from one TED patient revealed an improvement in EOM enlargement and edema following radiotherapy. This patient is a 55-year-old male with a 9-month history of TED. Before radiotherapy, coronal MRI demonstrated enlargement of the EOM on both T1- and T2-weighted imaging, along with increased T2 signal intensity **(A,B)**. Six months after radiotherapy, this patient showed a decrease in EOM enlargement and T2 signal intensity **(C,D)**.

This cohort included 10 patients with a CAS of 2 and 3 patients with a CAS of 1 but showing active inflammation on MRI at baseline. The remaining 37 patients had CAS values greater than 2. The mean CAS at baseline was 3.1 ± 0.99. Post-radiotherapy, the IOP, proptosis, and disease activity in TED patients significantly decreased. In addition, diplopia and eye motility restriction significantly improved, particularly with respect to downward and outward gaze. However, the synoptophore results revealed no significant change in either horizontal or vertical deviation (Table 2). Radiotherapy effectively alleviated edema in the EOMs while simultaneously reducing their overall volume. This was evident on MRI as a reduction in T2 signal intensity and muscle cross-sectional area (Figure 1). Statistical analysis revealed that the muscular index significantly decreased. In addition, visual acuity and refraction improved following radiotherapy, but the differences were not statistically significant. No radiotherapy-related complications were observed during the 6-month follow-up.

**TABLE 2:** Changes in ophthalmic clinical signs before and 6 months after radiotherapy in TED patients.

Ophthalmic clinical signs	Before radiotherapy	After radiotherapy	*P*-value
BCVA	0.91 ± 0.16	0.93 ± 0.16	0.15^a^
Spherical equivalent (D)	−0.83 ± 1.85	−0.78 ± 1.78	0.07 ^b^
IOP (mmHg)	20.29 ± 6.5	18.4 ± 5.21	**0.004** ^b^
CAS	3.1 ± 0.99	1.18 ± 0.72	**<0.0001** ^b^
Proptosis (mm)	17.32 ± 2.91	16.34 ± 2.96	**<0.0001** ^a^
Diplopia	2.5 ± 0.76	2.32 ± 0.98	**0.02** ^b^
Up	1.2 ± 1.37	1.04 ± 1.26	0.2^b^
Down	0.44 ± 0.86	0.18 ± 0.66	**0.002** ^b^
Abduction	0.08 ± 0.4	0.02 ± 0.14	0.5^b^
Adduction	0.76 ± 1.08	0.5 ± 0.93	**0.003** ^b^
Horizontal deviation (PD)	6.83 ± 8.5	7.67 ± 9.44	0.24^b^
Vertical deviation (PD)	11.93 ± 10.85	12.4 ± 11.75	0.75^b^
Muscular index	0.17 ± 0.03	0.15 ± 0.03	**<0.0001** ^a^

TED, thyroid eye disease; BCVA, best corrected visual acuity; IOP, intraocular pressure; CAS, clinical activity score; VISA, Vision, inflammation, strabismus, and appearance; PD, prism diopter.

^a^Paired Student’s *t*-test;

^b^Wilcoxon signed-rank test. Statistically significant *P*-values (<0.05) are highlighted in bold.

### Comparison of corneal morphological parameters between NCs and TED patients

We compared the corneal morphological parameters between NCs and TED patients. The results indicated that patients with TED presented greater flat (K1 front), steep (K2 front) and maximum keratometry (Kmax sagittal front) values on the anterior corneal surface, whereas both the flat keratometry (K1 back) and astigmatism values (Astigmatism back) on the posterior corneal surface were smaller in TED patients (Table 3). These findings suggest that individuals with TED tend to have steeper corneas. The corneal apex and thinnest thickness in the TED group showed no significant differences compared to the NC group. The TED patients presented significantly greater values of Db, Dp, Da, D, and ISV, along with a lower ARTmax. These findings indicate that while the overall corneal thickness in patients with TED did not show significant changes, they presented a more uneven distribution of corneal thickness and exhibited more irregular corneal morphology.

**TABLE 3:** Corneal morphological parameters in normal controls and TED patients before and 6 months after radiotherapy.

Corneal biomechanical parameters	NC	TED		NC versus TED (before RT)	Before RT versus After RT
		**Before RT**	**After RT**	***P*-value**	***P*-value**
K1 Front (D)	42.58 ± 1.35	43.28 ± 1.08	43.27 ± 1.07	**0.01** ^a^	0.61^c^
K2 Front (D)	43.56 ± 1.32	44.25 ± 1.1	44.26 ± 1.09	**0.01** ^b^	0.42^c^
Astigmatism front (D)	0.97 ± 0.72	0.97 ± 0.71	0.98 ± 0.75	0.87^a^	0.99^c^
K1 back (D)	−6.13 ± 0.24	−6.23 ± 0.19	−6.12 ± 0.75	**0.03** ^a^	0.21^c^
K2 back (D)	−6.49 ± 0.22	−6.52 ± 0.23	−6.53 ± 0.26	0.68^a^	0.22^c^
Astigmatism back (D)	0.36 ± 0.12	0.29 ± 0.18	0.41 ± 0.66	**0.03** ^a^	**0.01** ^c^
Kmax sagittal front (D)	44.14 ± 1.39	45.16 ± 1.26	45.47 ± 1.98	**0.001** ^b^	0.18^c^
Corneal thickness at the apex (mm)	560.8 ± 33.17	548.9 ± 32.37	549.6 ± 32.58	0.12^b^	0.74^d^
Thinnest corneal thickness (mm)	557.9 ± 32.74	543.6 ± 32.24	543.5 ± 32.28	0.08^a^	0.76^c^
ARTmax	464 ± 58.74	421.9 ± 100.5	417.1 ± 93.69	**0.04** ^b^	0.9^c^
Df	0.25 ± 0.81	0.01 ± 1.13	−0.05 ± 1.21	0.23^a^	0.18^c^
Db	0.27 ± 0.73	1.13 ± 1.28	0.83 ± 1.19	**0.001** ^b^	**0.01** ^c^
Dp	0.55 ± 0.79	0.97 ± 1.14	1.13 ± 2.12	**0.04** ^a^	0.99^c^
Dt	−0.51 ± 0.86	−0.13 ± 0.86	−0.11 ± 0.88	0.06^b^	0.48^d^
Da	0.22 ± 0.54	0.61 ± 0.92	0.65 ± 0.86	**0.04** ^b^	0.9^c^
D	0.81 ± 0.43	1.32 ± 0.97	1.2 ± 0.9	**0.001** ^a^	0.052^c^
ISV	14.8 ± 5.32	17.82 ± 6.27	19.74 ± 15.96	**0.03** ^a^	0.89^c^
IVA	0.12 ± 0.05	0.24 ± 0.68	0.17 ± 0.2	0.06^a^	0.51^c^
KI	1.02 ± 0.02	1.03 ± 0.04	1.03 ± 0.05	0.66^b^	0.53^c^
CKI	1.01 ± 0.006	1.01 ± 0.008	1.01 ± 0.012	0.32^a^	>0.999^c^
IHA	5.6 ± 4.65	6.37 ± 5.71	6.56 ± 5.57	0.71^a^	0.71^c^
IHD	0.01 ± 0.006	0.01 ± 0.008	0.02 ± 0.016	0.61^a^	0.98^c^

TED, thyroid eye disease; NC, normal control; RT, radiotherapy; K1, flat keratometry; K2, steep keratometry; Kmax, maximum keratometry; ARTmax, Ambrosio’s Relational Thickness maximum; Df, deviation from normality of the front elevation; Db, deviation from normality of the back elevation; Dp, deviation from normality of pachymetric progression; Dt, deviation from normality of the corneal thinnest point; Da, deviation from normality of relational thickness; D, overall deviation; ISV, index of surface variance; IVA, index of vertical asymmetry; KI, keratoconus index; CKI, center keratoconus index; IHA, index of height asymmetry; IHD, index of height decentration.

*^a^*Man-Whitney test;

*^b^*Unpaired Student’s *t*-test;

*^c^*Wilcoxon signed-rank test;

*^d^*Paired Student’s *t*-test. Statistically significant *P*-values (<0.05) are highlighted in bold.

**FIGURE 2:**
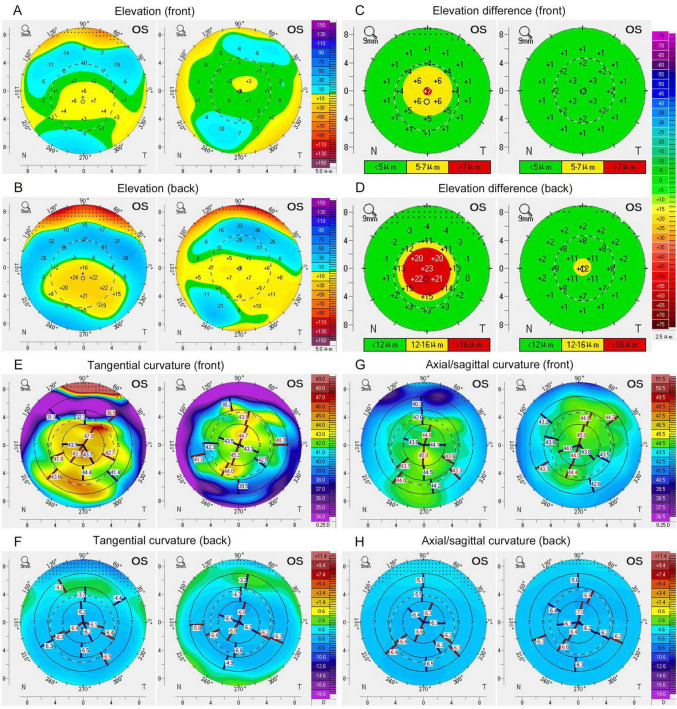
Corneal morphological changes in the TED patient at baseline and 6-month follow-up. Changes in corneal elevation **(A,B)** and elevation differences **(C,D)** were observed on both the anterior and posterior surfaces of the cornea following radiotherapy. Additionally, alterations in the corneal tangential curvature **(E,F)** and axial/sagittal curvature **(G,H)** were noted on both the anterior and posterior surfaces after radiotherapy. Left, before radiotherapy; Right, 6 months after radiotherapy.

We also compared the changes in corneal morphological parameters in TED patients at baseline and 6 months post-radiotherapy. Following radiotherapy, a reduction in the corneal elevation difference was observed, along with a significant change in in both anterior and posterior corneal surface curvature (Figure 2). Statistical analysis revealed a decrease in Db and an increase in astigmatism of the posterior surface (Astigmatism back), both showing statistically significant differences (Table 3). These findings suggest that radiotherapy can improve the irregularity of corneal morphology and alter corneal curvature.

### Comparison of corneal biomechanical parameters between NCs and patients with TED

We compared the corneal biomechanical parameters between NCs and TED patients. The results demonstrated that both the IOP and bIOP were significantly higher in patients with TED (Table 4). In the TED group, the A1 time was significantly prolonged, accompanied by a reduction in A1 velocity, whereas both the A2 and HC times were shorter. Given that IOP is a critical determinant of corneal biomechanics (23), we performed an adjustment with bIOP. Following this correction, the A2 time and peak distance remained significantly reduced, while the A2 velocity was notably increased. These findings collectively suggest diminished corneal hysteresis in TED patients. In addition, TED patients exhibited a significantly reduced WEML that remained statistically significant after correcting for bIOP, suggesting reduced globe movement in response to an air pulse and diminished orbital compliance in TED patients. Furthermore, TBI values were significantly higher in TED patients both before and after bIOP adjustment. This suggests a possible impairment in corneal biomechanical integrity.

**TABLE 4:** Corneal biomechanical parameters in normal controls and TED patients before and 6 months after radiotherapy.

Corneal biomechanical parameters	NC	TED		NC versus TED (before RT)		Before RT versus After RT	
		**Before RT**	**After RT**	***P*-value**	***P*-value^e^**	***P*-value**	***P*-value^e^**
IOP (mmHg)	16.75 ± 2.7	20.29 ± 6.5	18.4 ± 5.21	**0.0082^a^**	/	**0.0043^c^**	/
bIOP (mmHg)	16.07 ± 2.14	19.43 ± 5.74	17.75 ± 4.55	**0.0049^a^**	/	**0.0033^c^**	/
A1 time (ms)	7.78 ± 0.33	8.22 ± 0.85	7.98 ± 0.68	**0.019^a^**	0.274^e^	**0.0033^c^**	1^e^
A1 velocity (m/s)	0.15 ± 0.02	0.13 ± 0.03	0.14 ± 0.03	**0.0054^b^**	1^e^	**0.013^c^**	1^e^
A2 time (ms)	22.65 ± 0.43	22.05 ± 0.69	22.32 ± 0.65	**<0.0001** ^a^	**0.004^e^**	**0.0003^c^**	0.593^e^
A2 velocity (m/s)	−0.28 ± 0.03	−0.29 ± 0.05	−0.29 ± 0.04	0.31^a^	**0.001^e^**	0.77^d^	1^e^
HC time (ms)	18.04 ± 0.54	17.74 ± 0.46	17.87 ± 0.53	**0.0096^b^**	0.249^e^	0.10^c^	1^e^
Peak distance (mm)	4.89 ± 0.33	4.81 ± 0.49	4.88 ± 0.45	0.49^a^	**0.011^e^**	0.37^c^	0.703^e^
HC deformation amplitude (mm)	1.07 ± 0.12	1.03 ± 0.16	1.07 ± 0.17	0.14^b^	0.106^e^	**0.0065^d^**	1^e^
HC deflection amplitude (mm)	0.87 ± 0.11	0.86 ± 0.16	0.89 ± 0.16	0.88^a^	0.003^e^	0.17^d^	1^e^
WEML (mm)	0.31 ± 0.09	0.24 ± 0.06	0.28 ± 0.08	**0.0023^a^**	**0.017^e^**	**0.0002^c^**	**0.014^e^**
DA ratio	4.39 ± 0.45	4.24 ± 0.64	4.45 ± 0.66	0.10^b^	0.536^e^	**0.021^d^**	1^e^
Integrated radius (mm)	8.62 ± 1.03	8.6 ± 1.37	8.98 ± 1.63	0.55 ^b^	0.128^e^	0.078^c^	1^e^
SP A1	113.1 ± 16.48	118.9 ± 17.42	111.8 ± 16.71	0.12^b^	1^e^	**0.01^c^**	0.619^e^
CBI	0.04 ± 0.13	0.07 ± 0.21	0.14 ± 0.31	0.93^a^	0.91^e^	0.056^c^	0.846^e^
TBI	0.16 ± 0.14	0.39 ± 0.3	0.33 ± 0.24	**0.0002^a^**	**0.001^e^**	0.17^c^	0.903^e^

TED, thyroid eye disease; NC, normal control; RT, radiotherapy; HC, highest concavity; WEML, maximum whole eye movement length; WEMT, maximum whole eye movement time; DA ratio, deformation amplitude ratio at 2 mm; bIOP, biomechanically corrected intraocular pressure; SP A1, stiffness parameter A1; CBI, corvis biomechanical index; TBI, tomographic and biomechanical index.

*^a^*Man-Whitney test;

*^b^*Unpaired Student’s *t*-test;

*^c^*Wilcoxon signed-rank test;

*^d^*Paired Student’s *t*-test.

*^e^*Analysis of covariance with bIOP as covariates. Statistically significant *P*-values (<0.05) are highlighted in bold.

We also compared the changes in corneal biomechanical parameters in TED patients at baseline and 6 months post-radiotherapy. After radiotherapy, both the IOP and bIOP significantly decreased. In addition, there was a notable decrease in A1 time and SP-A1, along with an increase in A1 velocity, A2 time, HC deformation amplitude, and DA ratio. However, these changes became insignificant following bIOP adjustment. Furthermore, a significant increase in WEML was observed, which remained statistically significant after bIOP correction, suggesting that radiotherapy also improved orbital compliance in TED patients (Table 4).

### Correlation between the changes in clinical data and the corneal morphological and biomechanical parameters

Statistical analyses were conducted to assess potential correlations between clinical data and corresponding changes in corneal morphological and biomechanical properties. In terms of the corneal morphological changes following radiotherapy, proptosis was found to be negatively correlated with Db, suggesting that the improvement in proptosis may have an impact on corneal morphological changes after radiotherapy (Table 5).

**TABLE 5:** Correlation analyses between the changes in clinical data and the corneal morphological and biomechanical parameters before and 6 months after radiotherapy in TED patients.

Changes in corneal parameters	Changes in IOP		Changes in CAS		Changes in proptosis		Changes in diplopia		Changes in muscular index	
	***r*-value**	***P*-value**	***r*-value**	***P*-value**	***r*-value**	***P*-value**	***r*-value**	***P*-value**	***r*-value**	***P*-value**
Astigmatism back	−0.14	0.33	0.027	0.85	0.09	0.54	−0.11	0.43	−0.15	0.29
Db	−0.18	0.2	0.12	0.39	−0.29	**0.038**	−0.068	0.64	−0.10	0.49
A1 time	0.98	**<0.0001**	−0.007^a^	0.959^a^	0.298^a^	**0.038^a^**	0.178^a^	0.222^a^	0.209^a^	0.15^a^
A1 velocity	−0.5	**0.0002**	−0.047^a^	0.747^a^	−0.063^a^	0.667^a^	0.084^a^	0.564^a^	0.221^a^	0.126^a^
A2 time	−0.84	**<0.0001**	−0.049^a^	0.741^a^	−0.326^a^	**0.022^a^**	−0.019^a^	0.896^a^	−0.066^a^	0.65^a^
HC deformation amplitude	−0.73	**<0.0001**	−0.015^a^	0.916^a^	−0.247^a^	0.088^a^	−0.058^a^	0.694^a^	−0.036^a^	0.807^a^
WEML	−0.37	**0.008**	−0.175^a^	0.23^a^	−0.233^a^	0.107^a^	0.163^a^	0.262^a^	−0.183^a^	0.209^a^
DA ratio	−0.58	**<0.0001**	−0.211^a^	0.146^a^	−0.175^a^	0.23^a^	0.106^a^	0.467^a^	0.013^a^	0.931^a^
SP A1	0.29	**0.04**	0.004^a^	0.98^a^	0.265^a^	0.066^a^	−0.214^a^	0.14^a^	0.014^a^	0.926^a^

TED, thyroid eye disease; IOP, intraocular pressure; CAS, clinical activity score; Db, deviation from normality of the back elevation; HC, highest concavity; WEML, maximum whole eye movement length; WEMT, maximum whole eye movement time; DA ratio, deformation amplitude ratio at 2 mm; SP A1, stiffness parameter A1.

^a^Correlation analysis with adjustment for IOP. Statistically significant *P*-values (<0.05) are highlighted in bold.

For the biomechanical parameters, the results demonstrated that IOP was positively correlated with A1 time and SP-A1, along with negatively correlated with A1 velocity, A2 time, HC deformation amplitude, and the DA ratio. Additionally, the IOP was negatively correlated with the WEML. These results indicate that the reduction in IOP may be one of the reasons for the improvement in corneal hysteresis and orbital compliance. Besides, the correlation analysis after IOP adjustment revealed a positive correlation between proptosis and A1 time, as well as a negative correlation between proptosis and A2 time, indicating that improvements in proptosis may contribute to improved corneal hysteresis. Statistical analysis revealed no significant correlations between changes in CAS, diplopia, or muscular index and alterations in corneal morphology or biomechanics (Table 5).

## Discussion

At present, research on corneal morphology and biomechanics in TED remains limited. Only a few studies have reported the effects of surgical and medical interventions on corneal biomechanical properties in TED patients (9, 15, 22). There is a lack of data regarding changes in corneal morphology and biomechanical parameters following radiotherapy in patients with TED. In this study, patients with myopathic TED were found to have steeper corneas and more irregular corneal morphologies compared to NCs. After radiotherapy, improvements in irregular corneal morphology and changes in corneal curvature were observed. Additionally, radiotherapy can effectively improve corneal hysteresis and orbital compliance in TED patients. Furthermore, correlation analyses indicated that reductions in IOP and proptosis contribute to the improvements of corneal hysteresis and orbital compliance following radiotherapy in TED patients.

TED is classified into two subtypes. Approximately two-thirds of TED patients exhibit adipose tissue proliferation, which manifests as proptosis, upper eyelid retraction, and ocular exposure (24). The remaining one-third exhibit a myopathic phenotype, characterized by restricted ocular movement and diplopia (25, 26). In this study, individuals with myopathic TED demonstrate steeper corneas, a more uneven distribution of corneal thickness, and exhibit more irregular corneal morphology, which is generally consistent with previous research (16). These corneal changes likely result from biomechanical effects secondary to EOM enlargement and restriction in myopathic TED. The increased muscular tension induces characteristic topographic changes, including peripheral flattening with central steepening, which consequently elevates the TBI and potentially increases the risk of keratoconus development. Therefore, regular longitudinal evaluation of corneal morphology and biomechanics in myopathic TED patients is recommended, especially those with additional ectasia risk factors.

For the corneal biomechanical parameters, our study identified significant reduced corneal hysteresis and orbital compliance in patients with myopathic TED. The ocular biomechanical properties of patients with TED can vary depending on the activity and severity of the condition. Inflammatory cytokine-mediated activation of keratocytes during active disease stages stimulates the production of matrix metalloproteinases, leading to stromal degradation. Additionally, elevated tear film osmolarity plays a role in these changes. Collectively, these mechanisms contribute to viscoelastic alterations, resulting in alterations in corneal hysteresis (10, 27, 28). As the disease progresses, pathological changes may occur, including the enlargement of EOMs, proliferation of orbital adipose tissue, and fibrosis of orbital connective tissue. In addition, the deposition of GAG, including chondroitin sulfate and hyaluronan, can lead to orbital interstitial edema. These factors collectively increase intraorbital pressure, which in turn contribute to elevated WEML by reducing orbital compliance (11, 12, 29, 30). Moreover, the direct mechanical compression of the eye globe by the enlarged EOMs further exacerbates these biomechanical changes (16).

Currently, radiotherapy has been widely adopted as a standard second-line therapeutic approach for TED, particularly for the myopathic type. Radiotherapy can induce lymphocyte apoptosis, thereby disrupting the inflammatory cycle and subsequently decreasing inflammation (31). Additionally, radiotherapy can inhibit fibroblast differentiation and reduce GAG accumulation, which in turn decreases the volume of EOM. This reduction can alleviate muscular tension and improve restricted ocular motility (32, 33). A retrospective study revealed that radiotherapy may induce minor flattening of the central cornea and a slight improvement in the surface asymmetry index in TED patients, although these changes were not statistically significant (16). In this study, observed changes in corneal morphology and curvature were observed, and these changes are likely attributable to the reduction in EOM volume and muscle tension following radiotherapy. The cornea is a crucial component of vision, and radiotherapy-induced alterations in corneal curvature may have a potential impact on visual function. Despite no change was observed in visual acuity and refraction following radiotherapy, the treatment might still impact visual quality by affecting higher-order aberrations. Further research is needed to fully elucidate these effects. In addition, we performed the first comparison of ocular biomechanics in TED patients before and after radiotherapy, revealing significant improvements in orbital compliance after radiotherapy. These effects may be attributed to radiotherapy-induced inhibition of radiation-sensitive lymphocyte activation, suppression of fibroblast proliferation, and reduction in GAG secretion, collectively leading to decreased orbital pressure and improved orbital compliance.

Correlation analyses revealed that reductions in proptosis contribute to improvements in ocular biomechanics. In myopathic TED, the primary feature is EOM enlargement without significant adipose tissue expansion (25). we speculate that the improvement in EOM enlargement due to radiotherapy, leading to a reduction in orbital contents, alleviation of globe protrusion, and decreased retrobulbar resistance, which consequently results in improved corneal hysteresis and orbital compliance.

The elevation of IOP in TED patients is linked to several factors, including increased episcleral venous pressure resulting from elevated intraorbital pressure, compression by enlarged and fibrotic EOMs, and increased deposition of GAG in the trabecular meshwork, which obstructs aqueous humor outflow (34–36). Radiotherapy reduces orbital inflammatory infiltration by inducing lymphocyte apoptosis and decreases intraorbital pressure, which subsequently lowers IOP (31). Additionally, radiotherapy may directly affect the IOP regulation system, including the trabecular meshwork and ciliary body (37). Higher IOP corresponds with increased corneal resistance and decreased WEML, as measured by the Corvis ST (23). Our study revealed a positive correlation between a reduction in IOP following radiotherapy and improvements in corneal hysteresis and orbital compliance, which is consistent with the findings of previous studies (9, 23). Furthermore, our study revealed significant reductions in A1 time and SP-A1, accompanied by increases in A1 velocity, A2 time, HC deformation amplitude, and DA ratio after radiotherapy. However, these alterations became non-significant after bIOP adjustment, suggesting that IOP may be a primary factor influencing these corneal biomechanical changes.

This study has several limitations. First, this study included only TED patients of the myopathic type, limiting the applicability of these findings to adipose type TED cases. Second, the follow-up period of 6 months was relatively short, necessitating extended observation periods in future studies. Third, progressive fibrotic remodeling may significantly influence corneal morphology and biomechanics in TED. While radiotherapy effectively reduces orbital inflammation and EOM volume, its specific effects on fibrotic processes and their subsequent impact on corneal properties require further investigation. Fourth, TED-associated changes in tear film dynamics could potentially affect corneal parameters. The possible modulation of tear film stability by radiotherapy and its subsequent effects on corneal properties need additional study to fully understand the treatment’s comprehensive ocular effects.

## Conclusion

In conclusion, our study revealed that patients with myopathic TED exhibited steeper and more irregular corneas. Biomechanically, these patients demonstrated higher TBI, reduced corneal hysteresis and orbital compliance. Our findings suggest that myopathic TED patients with additional pre-existing risk factors for corneal ectatic disorders, particularly keratoconus, should undergo longitudinal assessments of their corneal morphological and biomechanical properties. Following radiotherapy, improvements were observed in both corneal irregularity and curvature, along with significant enhancements in ocular biomechanics. Our analysis revealed significant correlations between proptosis, IOP, and corneal biomechanical parameters. These findings suggest that ocular biomechanical properties may serve as potential biomarkers for evaluating treatment efficacy in TED management.

## Data Availability

The original contributions presented in this study are included in this article/supplementary material, further inquiries can be directed to the corresponding author.
